# The Semantics of Natural Objects and Tools in the Brain: A Combined Behavioral and MEG Study

**DOI:** 10.3390/brainsci12010097

**Published:** 2022-01-12

**Authors:** Elisa Visani, Davide Rossi Sebastiano, Dunja Duran, Gioacchino Garofalo, Fabio Magliocco, Francesco Silipo, Giovanni Buccino

**Affiliations:** 1 Neurophysiology Unit, Fondazione IRCCS Istituto Neurologico Carlo Besta, Via Celoria 11, 20133 Milan, Italy; Elisa.Visani@istituto-besta.it (E.V.); davide.rossi@istituto-besta.it (D.R.S.); Dunja.Duran@istituto-besta.it (D.D.); 2 Division of Neuroscience, IRCCS San Raffaele Scientific Institute, University San Raffaele, Via Olgettina 60, 20132 Milan, Italy; g.garofalo@docenti.unisr.it; 3 Centro Psico-Sociale di Seregno—Azienda Socio-Sanitaria Territoriale di Vimercate, 20871 Vimercate, Italy; fabio.magliocco@asst-brianza.it; 4 Dipartimento di Scienze Mediche e Chirurgiche, University “Magna Graecia” of Catanzaro, Viale Salvatore Venuta, 88100 Germaneto, Italy; silipo@unicz.it

**Keywords:** semantics, object representations, behavioral responses, MEG, embodiment, beta rhythm

## Abstract

Current literature supports the notion that the recognition of objects, when visually presented, is sub-served by neural structures different from those responsible for the semantic processing of their nouns. However, embodiment foresees that processing observed objects and their verbal labels should share similar neural mechanisms. In a combined behavioral and MEG study, we compared the modulation of motor responses and cortical rhythms during the processing of graspable natural objects and tools, either verbally or pictorially presented. Our findings demonstrate that conveying meaning to an observed object or processing its noun similarly modulates both motor responses and cortical rhythms; being natural graspable objects and tools differently represented in the brain, they affect in a different manner both behavioral and MEG findings, independent of presentation modality. These results provide experimental evidence that neural substrates responsible for conveying meaning to objects overlap with those where the object is represented, thus supporting an embodied view of semantic processing.

## 1. Introduction

Classically, semantics refers to our capacity to attribute meaning to the events and entities (such as objects, words, feelings, and so on) that we experience during our lifespan and organize in a symbolic system. Language is the symbolic system that we use to represent this knowledge about the world, but how this knowledge is organized in the brain and how it is related to the real world is a matter of debate within the neuroscientific literature. In recent times, it has been proposed that the speakers understand linguistic material thanks to the recruitment of those sensory, motor, and even emotional systems involved in experiencing the content expressed by that linguistic material [1,2,3,4,5,6,7]. This approach contrasts with a more classical one, claiming language as an amodal function, that is completely disentangled from those sensorimotor systems normally involved in experiencing its content [8,9,10,11]. Indeed, these two approaches are not mutually exclusive, and some authors have tempted to combine the two views. In this perspective, authors do not deny a potential role of sensory, motor, and emotional systems in building up concepts. As for objects, their concepts are stored in brain areas distinct from those where individuals experience the different features of objects [12,13,14,15,16]. 

Within this general framework, the central claim is that information about the features of an object—such as its form, its size, and the manner in which we act upon it—is stored in our sensorial, motor, and emotional systems [16]. For instance, it has been demonstrated that words related to odorants (e.g., cinnamon) activate the olfactory system [17], whereas words related to taste (e.g., salt) activate the gustatory one [18] and words related to emotions activate the corresponding area (e.g., disgust, [19]). However, despite the recruitment of sensorimotor and even emotional areas, possibly related to coding specific features of the objects expressed by the nouns, the meaning of the noun per se is not coded in those areas, but rather in distinct high-order, linguistic regions, the so-called semantic hubs [13,16,20]. In other words, this evidence does not rule out the possibility that, despite the involvement of the cortex used to experience the sensory or motor content, the mechanism (and possibly the areas) allowing us to attribute the meaning could be different [13,16,21]. 

A further point of interest is that, in the current literature, observed objects seems to be differently analyzed from their corresponding nouns. There is a general agreement that two visual streams subserve the processing of objects when observed [22,23,24]. When individuals have to interact with objects, the dorsal stream, including frontal and parietal areas, is mainly involved. This stream is devoted to sensorimotor transformation that make possible the choice of the most appropriate motor program to act upon the observed object. This implies that, within the dorsal stream, there are represented both the object features used to guide actions (e.g., the size, the orientation), and the actions usually performed upon an object (e.g., [25,26]). It has been demonstrated that the dorsal stream is anatomically and functionally composed by two circuits, named dorso-dorsal and dorso-ventral stream, in which natural objects and tools are represented, respectively [27,28,29,30]. Accordingly, the representation of the actions linked to these categories of objects are also coded in these circuits. Manipulative actions (such as power and precision grips, simple actions like key pressing used in the present study to provide motor responses, up to reach-to-grasp actions) specifically devoted to interact with natural objects are represented in the dorso-dorsal stream, while actions for use (such as to grasp the hammer to drive the nails) are represented in the dorso-ventral one, which also seems accountable for the processing of sensorimotor information based on long-term object representation [28,29,30]. Despite this different representation, current literature claims that the recognition of an object is subserved by the ventral stream as described by pivotal studies [22] including some specific temporal areas (e.g., lateral occipital temporal cortex, anterior and inferotemporal regions). In further support of this view, clinical findings showed that following a damage in the temporal lobe, patients lose the ability to recognize an object, while after damage to the parietal cortex, they lose the ability to use objects properly [28,31,32,33,34,35]. Indeed, these two streams are not completely segregated but they rather interact to update our functional knowledge and our capacity to interact online with an object [25,28,36,37,38,39,40,41].

Tools are a special class of graspable objects for humans. The study of tools is interesting since they have an associated functional use that involves a particular modality of interaction with the object, rather than just the feature to be grasped, as natural objects have [28]. Furthermore, humans use tools in different contexts, thus requiring a generalization process and the conceptual knowledge of their use [33]. Functional neuroimaging studies focusing on tools have demonstrated that their use elicits an activation of many distinct brain areas, including the left supramarginal gyrus (SMG) [42,43,44,45,46,47,48,49,50,51], the ventral premotor cortex (PMv) [26,52,53], the left inferior frontal gyrus (IFG) bordering pars opercularis [33], and the left insula [43]. Overall, these studies show that tools are represented in circuits distinct from those when natural objects are represented. Specifically, tools seem to be represented in a fronto-parietal circuit corresponding to ventro-dorsal subdivision of the dorsal stream [27,29]. Note that, as previously mentioned, in this part of the dorsal stream the specific actions involved in the specific use of the objects are also represented. Moreover, this different representation is possible already present in non-human primates [54]. 

To sum up, current literature seems to support the view that the observation of graspable natural objects and graspable tools lead to the activation of different sectors within the dorsal stream, in which also the congruent actions are represented, while the processing and understanding of nouns expressing objects in the same categories lead to activation of shared semantic hubs. These areas are therefore distinct from those where objects are coded and where actions necessary to interact with them are represented.

Indeed, for natural objects, some recent studies have suggested that the verbal labels and the observed objects share similar semantic mechanisms [2,55,56,57,58,59,60,61,62]. In two behavioral studies, which used a similar paradigm as in the present study, participants gave slower motor responses for natural graspable objects and nouns as compared to non-graspable ones [56,57]. The authors proposed that when participants are engaged in two different tasks, i.e., the object processing (either pictorially or verbally presented) and the preparation of motor responses, the motor system is involved in both tasks and, therefore, there is a competition for neuronal resources, leading to a slowing down of motor responses. 

In the present study, we directly compared the modulation of the motor system during the processing of natural graspable objects either verbally or pictorially presented, and graspable tools, also either verbally or pictorially presented. In line with the embodiment approach, since natural graspable objects and tools have distinct motor representations in the brain, these two object categories should lead to a different modulation of the motor system, regardless of the presentation modality. Namely, graspable natural objects should recruit the most dorsal sector of the dorsal stream, while tools should recruit the ventral sector of the dorsal stream. On the contrary, if the processing of nouns involves areas distinct from those where the corresponding objects are motorically represented, then a different modulation could be still potentially found for observed objects, but it appears unlikely for nouns, since the nouns of objects are coded in specific hubs distinct from the regions where natural objects and tools, respectively, are motorically represented [13,16,20]. In the present study, we addressed this issue with a go/no-go task already used in previous studies of our group [56,63], where participants gave their responses when stimuli where real words and/or objects and had to refrain from responding when stimuli were meaningless (scrambled images or pseudowords). Responses were asked at 150 ms from stimulus onset, since previous studies have shown that the motor system is early recruited just 150–170 ms after the visual or auditory presentation of stimuli with a specific motor content [64,65,66,67,68,69,70,71]. We replicated the behavioral task in a Magnetoencephalography (MEG) study, looking at the modulation of the cortical Beta rhythm during the semantic processing of natural objects and tools, presented either as nouns or as images. Beta band oscillations are the predominant rhythm originating in the motor cortex with a typical pattern of suppression and rebound during movement [72]. Beta suppression, or desynchronization (event-related desynchronization, ERD), starts several hundred milliseconds before movement onset in self-paced or externally cued movements and becomes maximal around the time of movement execution. ERD and subsequent synchronization (event-related synchronization, ERS) were largely adopted to study the neural correlates of action observation [73,74], motor imagery [75,76,77] and action related language [63,78]. In sum, an increase suppression of beta rhythm expresses a condition where the motor system is more prompt to generate a motor response, while a weaker suppression of beta rhythm expresses a condition where the motor system is less prompt for action. 

In the present study, magnetic ERD/ERS in the beta band has been exploited to reveal the neural correlates of object observation and noun processing and the underlying neurophysiological mechanisms of motor responses. As foreseen by embodiment, we expected a different modulation of motor responses as well as beta rhythm, during the processing of natural graspable objects and tools, respectively, given their different motor representation in the brain. This independent of the presentation modality (nouns and pictures) of the two kinds of stimuli. In other words, we aimed at assessing whether beta rhythm suppression is also sensitive to graspable natural objects (presented as picture and nouns), as it is to congruent manipulative hand actions. Note that we used as a motor response just a manipulative action (i.e., a simple key pressing, most likely involving neural structures actually used to interact with natural graspable objects) and not an action for use as those required when interacting with graspable tools [27,28,29,30]. Since the cortical circuitry involved in generating the motor response required by the behavioral task was also involved, at the same time, in the semantic processing of the natural graspable objects, we expected a weaker suppression of beta rhythm, in parallel with slowing down of motor responses, during the processing of this object category as compared to tools, regardless of the presentation modality.

## 2. Materials and Methods

### 2.1. Experiment 1—Behavioral Study

#### 2.1.1. Participants

In total, 28 volunteers (18 females, mean age = 22 years old and 5 months, Std. Dev. = 3.2) took part in the behavioral experiment. All participants were 18 years or older prior to participating, they gave their informed consent, accordingly with the ethical standards of the Declaration of Helsinki. Exclusion criteria were formal education in linguistics, the presence of neurological or psychiatric disorders and the use of drugs affecting the central nervous system. The study was approved by the Ethics Committee of the University “Magna Graecia” of Catanzaro (approval number: 2012.40, date of approval: November 2012) and complied with the ethical standards of the Italian Psychological Society (AIP, see http://www.aipass.org/node/26, accessed on 21 November 2020) as well as the Italian Board of Psychologists (see http://www.psy.it/codice_deontologico.html, accessed on 21 November 2020). All participants were right-handed, according to the Edinburgh Handedness Inventory [79], had normal or corrected-to-normal vision, and were native Italian speakers.

#### 2.1.2. Apparatus, Procedure and Stimuli

The experiment was carried out in a sound-attenuated room, dimly illuminated by a halogen lamp directed toward the ceiling. Participants sat comfortably in front of a PC screen (LG 22′′ LCD, 1920 × 1080 pixel resolution and 60 Hz refresh rate). The eye-to-screen distance was set at 60 cm.

The experiment used a go/no-go task, in which participants were requested to respond to real nouns and images of objects and refrain from responding when presented stimuli were pseudowords and scrambled images. The experiment session consisted of 1 practice block and 1 experimental block. In the practice block, participants were presented with 16 stimuli (4 images of natural objects or tools, 4 scrambled images, 4 nouns of natural objects or tools, and 4 pseudowords) which were not used in the experimental block. During the practice block, participants received feedback (“ERROR”) after giving a wrong response (i.e., responding to a meaningless or refraining from responding to a real item), as well as for responses given prior to go signal presentation (“ANTICIPATION”), or later than 1.5 s (“YOU HAVE NOT ANSWERED”). In the experimental block, each stimulus was randomly presented twice with the constraint that no more than three items of the same kind (verbal, visual) or referring to objects of the same category (graspable natural object, tools, meaningless) could be presented on consecutive trials. No feedback was given to participants. Thus, the experiment, which lasted about 20 min, consisted of 160 go trials (80 nouns, 50% natural graspable object nouns and 50% tools nouns, plus 80 images of objects, 50% natural graspable objects and 50% tools) and 160 no-go trials (80 pseudowords plus 80 scrambled images), and 16 practice trials, for a total of 336 trials. To sum up, the experiment used a 2 × 2 repeated measures factorial design with Category (natural graspable objects, graspable tools) and Stimulus Type (nouns, photos) as within-subjects variables. 

Nouns in the 2 categories were matched for word length (mean values for nouns referring to natural objects and tools: 6.4 and 7.4; t = 0.049, *p* = 0.96), syllable number (mean values: 2.45 and 3.00; t = 0.018, *p* = 0.98) and written lexical frequency [mean values: 6.14 and 8.77 number of occurrences per million in CoLFIS (Corpus e Lessico di Frequenza dell’Italiano Scritto ~3.798.000 words)—Laudanna et al., 1995; t = 0.52, *p* = 0.60]. Pseudowords were built by substituting one consonant and one vowel in two distinct syllables of each noun (e.g., “sgalpillo” instead of “scalpello”). With this procedure, pseudowords contained orthographically and phonologically legal syllables for the Italian language. Hence, nouns and pseudowords were also matched for length. 

Images depicted 20 natural graspable objects and 20 tools. They were photos of real objects and not sketches. The scrambled images were built by applying Photoshop distorting graphic filters (e.g., blur and twist) to the photos depicting both natural graspable objects and graspable tools, so to make them unrecognizable and then meaningless. All photos and scrambled images were 440 × 440 pixels. The list of stimuli is reported in the Supplementary Materials.

In order to avoid any priming effect due to the presentation of the same item in different modalities, nouns and images in a specific category (e.g., graspable tools) never depicted the same item (for example, a graspable tool like “hammer” was presented as a noun but not depicted as an image; coherently a graspable tool like “axe” was presented as an image but not as a noun). 

**Figure 1:**
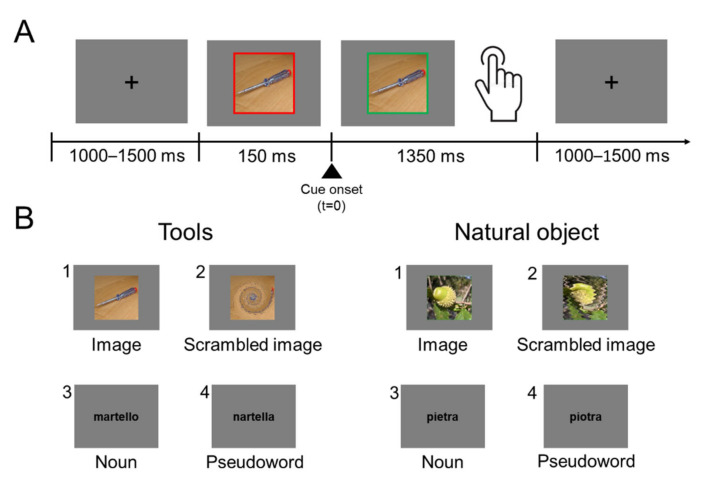
Experimental procedure. (**A**) Task timing: Participants were asked to fixate the center of the screen placed in front of them. Each trial started with the presentation of the stimulus surrounded by a red frame. After 150 ms the frame turned green and the participants were allowed to respond. Participants were instructed to respond only if the stimulus referred to a real tool or to a real natural graspable object. The trial ended when participants provided their responses or after 1350 ms if no response was given. (**B**) Stimuli examples: images (1), scrambled images (2), nouns (3) and pseudowords (4).

Each trial started with a black fixation cross (RGB coordinates = 0, 0, 0) displayed at the center of a grey background (RGB coordinates = 178, 178, 178). After a delay of 1000–1500 ms (in order to avoid response habituation), the fixation cross was replaced by a stimulus item, either a noun/pseudoword or an image/scrambled image. Note that the delay could be at any time between 1000 and 1500 ms. The verbal labels were written in black lowercase Courier New bold (font size = 24). Stimuli were centrally displayed and surrounded by a red (RGB coordinates = 255, 0, 0) 20 pixels-wide frame. The red frame changed to green (RGB coordinates = 0, 255, 0) 150 ms after the stimulus onset. The color change of the frame was the “go” signal for the response (Figure 1). Participants were instructed to give a motor response, as fast and accurate as possible, by pressing a key on a computer keyboard centered on participants’ body midline with their right index finger. They had to respond when the stimulus referred to a real object, and refrain from responding when it was meaningless. After the go signal, stimuli remained visible for 1350 ms or until participant’s responses. Stimulus presentation and response times (RTs) collection were controlled using the software package E-Prime 2.

#### 2.1.3. Data Analysis

Data analyses were performed using R 3.6.3 [80]. Practice trials were excluded from analysis. Participants’ RTs to real stimuli were analyzed. The RTs were measured from the “go” signal to the button pressing. Mean RTs of each participant were submitted to an rmANOVA, with Category (2 levels: natural graspable object and tool) and Stimulus type (2 levels: noun and image) as factors. 

### 2.2. Experiment 2—MEG Study

#### 2.2.1. Participants

In total, 15 volunteers (9 females, mean age = 26 years old, Std. Dev. = 2.0) were recruited for the experiment. All participants were 18 years or older prior to participating. All participants were right-handed, according to the Edinburgh Handedness Inventory (Oldfield, 1971), had normal or corrected-to-normal vision and were native Italian speakers. Exclusion criteria were formal education in linguistics, the presence of neurological or psychiatric disorders and the use of drugs affecting the central nervous system. The experiment was carried out in accordance with the ethical standards laid down in the 1964 Declaration of Helsinki and its later amendments. The study was approved by the Ethics Committee of Fondazione IRCCS Istituto Neurologico Carlo Besta of Milan (approval number: 47/2012; date of approval: November 2012) and the University “Magna Graecia” of Catanzaro (approval number: 2012.40) and complied with the ethical standards of the Italian Psychological Society (AIP, see http://www.aipass.org/node/26, accessed on 21 November 2020) as well as the Italian Board of Psychologists (see http://www.psy.it/codice_deontologico.html, accessed on 21 November 2020). Participants gave their written informed consent before being included in the study.

#### 2.2.2. Task

Participants were seated in the magnetically shielded room to perform the experiment. Stimuli and procedure were the same of the behavioral study, with the necessary adaptation required by the MEG setting used in the current study. Sixteen practice trials were used to train participants. To improve signal-to-noise ratio, the experiment consisted of two consecutive acquisitions in each of which 80 go trials (40 nouns, 50% natural object nouns and 50% tools nouns, plus 40 images of object, 50% natural objects and 50% tools) and 80 no-go trials (40 pseudowords plus 40 scrambled images) were presented, for a total of 320 experimental trials. In the two acquisitions, the presentation order of the stimuli was randomized. Hence, the MEG study used the same 2 × 2 repeated measures factorial design as the behavioral one. Stimulus presentation and RTs collection were controlled using the software package Stim2.

#### 2.2.3. MEG Data Acquisition and Pre-Processing

The MEG signals were acquired using a 306-channel whole head MEG system (Triux, Elekta Oy, Helsinki, Finland). Surface EMG signals were simultaneously recorded from pairs of electrodes placed bilaterally 2–3 cm apart over the belly of the right and left flexor and extensor of wrist. Signals were sampled at 1 kHz. Moreover, bipolar electro-oculographic (EOG) and electrocardiographic signals (ECG) were acquired.

The participant’s head position inside the MEG helmet was continuously monitored by five head position identification (HPI) coils located on the scalp. The locations of these coils, together with three anatomical landmarks (nasion, right and left preauriculars), and additional scalp points were digitized before the recording by means of a 3D digitizer (FASTRAK, Polhemus, Colchester, VT, USA). 

The raw MEG data were pre-processed off-line using the spatio-temporal signal-space separation method [81] implemented in the Maxfilter 2.2 (Elekta Neuromag Oy, Helsinki, Finland) in order to subtract external interference and correct for head movements and then band-pass filtered at 0.1–100 Hz.

Cardiac and ocular movement artifacts were removed using ICA algorithm based on EEGLAB toolbox [82] implemented in a custom-made MATLAB code (R2017b, Mathworks Inc., Natick, MA, USA), using ECG and EOG as reference. MEG data were divided into epochs ranging from 2.2 s before to 2.8 s after the stimulus onset. The epoch length was chosen by taking into account the reaction time and the motor activation defined by the EMG signal, including the return to baseline. Epochs with continuous muscular contraction and/or sensor jumps were excluded from further analysis. 

Finally, data epochs were grouped according to the four experimental conditions (natural and tools images, natural and tools words).

### 2.3. Data Analysis

#### 2.3.1. Sensors Analysis

Time–frequency representations (TFR) were computed across frequencies from 1 to 30 Hz (in 1 Hz steps) and time from −2 to 2.5 s (in 0.1 s steps) with a fixed frequency smoothing of 4 Hz. Desynchronization values were obtained as percent power change in beta band (15–30 Hz) calculated with respect to mean power in the −2 to −1 s before cue onset. Finally, for each participant, the most reactive β-band frequency (individual reactive frequency, IRF) was defined as the frequency at which the maximum desynchronization was found. 

#### 2.3.2. Source Analysis

Dynamic imaging of coherent sources (DICS) beamforming [83] was used to identify the spatial distribution in the frequency domain. The leadfield matrix was computed using realistically shaped single-shell volume conduction model based on template brain co-registered by means of digitized scalp points. Source model was obtained from a 5 mm resolution grid which covered whole brain volume. Source localizations was performed for the band IRF ± 1 Hz for a pre-stimulus baseline period (−1.2 to −0.5 s) and for a window of interest during stimulus presentation (0.5 to 1.2 s) using a common spatial filter based on the pooled data from both time intervals. Subject-specific relative power differences were grand-averaged and normalized to the MNI brain template. 

Source time-series were extracted using the linearly constraint minimum variance (LCMV) beamforming [84] with 5% regularization. Data were normalized to the MNI template to extract the source time-series on inferior parietal lobule and precentral and postcentral areas. Subsequently, as for the sensors data, we calculated the desynchronization in IRF ± 1 Hz band and averaged within regions. The ERD onset latency was defined as the first value leading to the minimum/maximum value and the ERD offset was defined as the first value returning to baseline. Finally, the desynchronization AUC was calculated between ERD onset and offset.

Both sensor and source data analysis were analyzed using custom Matlab (MATLAB 2017a, MathWorks, Inc., Natick, MA, USA) scripts based on SPM8 [85] and Fieldtrip toolboxes [86].

#### 2.3.3. Statistical Analysis

The RTs were compared using repeated measures ANOVA (rmANOVA) with the factor Category (tools, natural) and Stimulus type (images, nouns).

To preliminary explore the involvement of sensorimotor areas in the contralateral hemisphere on sensors, the non-parametric permutation test in combination with cluster-level statistics and multiple comparison correction implemented in Fieldtrip toolbox was applied. Post-hoc paired two-tailed *t*-tests were used to calculate the within-group difference between stimuli. 

Finally, to compare the beta ERD Area Under the Curves (AUCs) on source signals in terms of main effects and interactions, rmANOVA were performed with Category, Stimulus type and ROIs (inferior parietal lobule, precentral and postcentral areas) as factors. Statistical analyses were carried out using IBM SPSS, version 20 (SPSS Inc., Chicago, IL, USA). All data are expressed as mean ± standard errors of mean.

To compare TFR between different conditions in motor areas contralateral to the response hand, and to identify significant beta frequencies and time points, the non-parametric permutation test in combination with cluster-level statistics and multiple comparison correction implemented in Fieldtrip toolbox was applied. Post-hoc paired two-tailed *t*-tests were used to calculate the within-group difference between stimuli. All data are expressed as mean ± standard errors of mean. Statistical analyses were carried out using IBM SPSS, version 20 (SPSS Inc., Chicago, IL, USA). The RTs and beta ERD AUCs were compared using rmANOVA with the factor Category (tools, natural) and Stimulus type (images, nouns). 

## 3. Results

### 3.1. Experiment 1—Behavioral Study

Data were collected from twenty-eight participants. One participant was excluded from analysis since he/she performed 120 errors. All other participants performed the task well with few errors (Mean error rate: 5.3%, SD = 2.9). Error trials were checked, excluded without replacement and they were not analyzed further. RTs were calculated as the interval between cue onset and the key press on the computer keyboard.

Repeated measures ANOVA (rmANOVA) on RTs revealed the main effect of Category (F(1, 26) = 99.64; MSE = 382.94; *p* < 0.001). Slower RTs were obtained with natural graspable objects as compared with tools (714 ± 11.89 ms vs. 677 ± 11.55 ms). Responses to natural stimuli were slower than those to tools both for images (t(26) = 6.79, *p* < 0.0001) and nouns (t(26) = 5.41, *p* < 0.0001). Neither the main effect of Stimulus Type (F(1, 26) = 1.57, *p* = 0.221) nor the interaction (F(1, 26) = 0.76, *p* = 0.390) reached the statistical significance. Descriptive statistics are reported in Table 1.

### 3.2. Experiment 2—MEG Study

#### 3.2.1. Behavioral Data

Behavioral data from 15 participants replicated the results of Experiment 1. All subjects performed well with few errors (Mean error rate: 4.2%. SD = 2.3). RTs were calculated as the interval between cue onset and the EMG onset. rmANOVA on RTs showed a main effect of Category (F(1,14) = 22.18, MSE = 27,093.8, *p* < 0.001). RTs to natural stimuli were slower than those to tool stimuli both for images (Natural: 573.3 ± 11.61 ms; Tools: 536.3 ± 13.2 ms, t(14) = 3.612, *p* = 0.003) and nouns (Natural: 593.3 ± 18.9 ms; Tools: 545.9 ± 16.3 ms, t(14) = 3.877, *p* = 0.002). Neither the main effect of Stimulus Type nor the interaction reached the statistical significance. 

#### 3.2.2. MEG Data

Time-frequency analysis on sensors.

**Figure 2:**
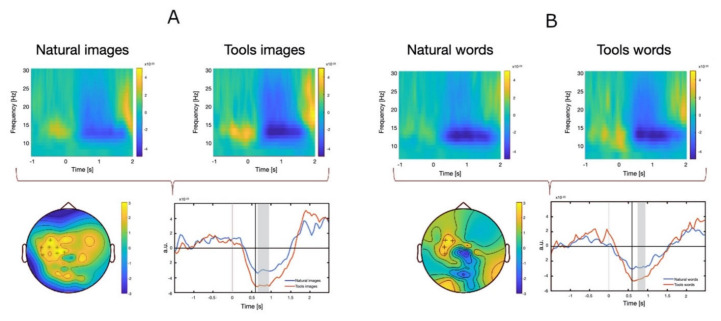
(**A**). Time-frequency representations (TFR) for each pair of stimulus type comparison ((**A**): natural vs. tools images; (**B**): natural vs. tools nouns). Upper panel. Mean TFR values of the sensors in the contralateral motor area for the different stimuli. Note the beta pattern of desynchronization (reduction of power) and synchronization (increase of power) more evident in the case of tools stimuli. Lower panel. On the left, map of significant difference in beta band averaged over the time interval between 0.6 and 0.9 s for images and between 0.7 and 0.9 s for nouns, Asterisks indicate *p* < 0.01, plus indicate *p* < 0.05. On the right, time course of beta band power modification for each stimulus type. Shadowed area indicates the time range where the difference was significant (*p* < 0.05).

The typical time-frequency pattern was observed in every subject and condition consisting in beta band desynchronization over the contralateral motor area immediately after stimulus onset followed by focal synchronization after movement execution. When comparing natural graspable objects and tools (both for images and nouns), statistical analysis revealed a significant difference in the interval between 0.5 and 1 s after stimulus onset in contralateral motor area. Specifically, a significant greater desynchronization was found for tools stimuli with respect to natural stimuli (Figure 2A,B). The difference was greater and more protracted in the case of visual stimuli as compared to nouns (Figure 2, bottom panels). No significant differences were found in the remaining comparison (natural object images vs. nouns, tool images vs. nouns).

#### 3.2.3. Source Analysis

**Figure 3:**
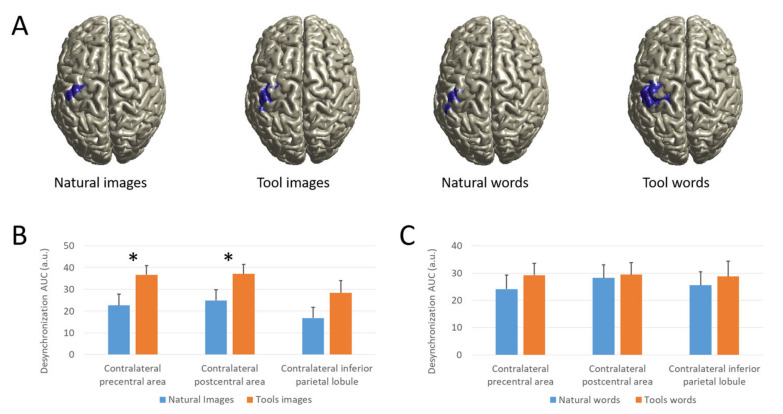
(**A**): Source analysis of beta activity. Source estimation projected onto the MNI template brain of grand-averaged power modulation obtained by contrasting −1.5 to −0.5 s vs. 0.5 to 1.5 s with respect to the cue onset in 15–25 Hz band for each condition. For illustrative purpose, only values greater than 80% of the maximum are shown. (**B**,**C**): Beta desynchronization AUC. Beta AUC values for natural and tools images (**B**) and nouns (**C**) condition. Note that the natural stimuli values are smaller than tools stimuli in both images and nouns condition in all areas, confirming the main effect of Category. Asterisk indicates significant difference in *t*-tests. Data are represented as mean±.

Cortical sources of beta power modulation by means of dynamic imaging of coherent sources (DICS) are illustrated in Figure 3A. Beta power modulations were most pronounced in contralateral pericentral regions, including pre-central, post-central and inferior parietal areas that we used as ROIs. Comparing the beta desynchronization in the area under the curve (AUC) of selected ROIs, rmANOVA showed a significant main effect of Category (F(1,14) = 11.586, *p* = 0.004) and ROIs (F(1.450,20.301) = 4.168, *p* = 0.041) and a trending towards significant interaction between Category and Modality (F(1,14) = 3.764, *p* = 0.073). Taking into account the signals for all natural object vs. tool stimuli, irrespective of modality, a significantly greater desynchronization AUC was found in each ROIs (precentral: t(14) = −2.683, *p* = 0.018; postcentral: t(14) = −3.641, *p* = 0.003; IPL: t(14) = −2.282, *p* = 0.039). Comparing separately the different modality, for the images the ERD AUC was significantly greater for the tools stimuli with respect to the natural stimuli in precentral (t(14) = −3.279, *p* = 0.005) and postcentral (t(14) = −3.597, *p* = 0.003) areas and almost significant in IPL (t(14) = −1.971, *p* = 0.069), whereas for nouns no significance was found (Figure 3B,C).

## 4. Discussion

The results of the present study are relevant for the current literature about the semantics of objects. The first element of interest is that observed graspable objects and verbal labels of the graspable objects (i.e., nouns) showed a similar modulation of the activity of the motor system as revealed both by RTs and Beta rhythm as measured by MEG. It is worth stressing that the beta rhythm is known to be generated in frontal and parietal areas.

For natural objects, participants gave slower motor responses compared to tools, regardless of the presentation modality. Since participants gave their hand motor responses with a simple manipulative action (i.e., key pressing) involving the same neural structures where graspable natural objects are represented and semantically processed (dorso-dorsal sector of the dorsal stream), these results can be interpreted as an interference effect, because the same neuronal resources were involved at the same time in attributing meaning to the object and to perform motor response required by the task. Hence, participants paid a cost showing a slowing down of their motor responses. In the present study, this modulation occurred with natural graspable objects, either verbally or visually presented.

A similar pattern of motor responses has been found in a previous study [56] that compared seen and verbally labelled natural graspable and non-graspable objects, with a slowing down of RTs with graspable objects as compared to non-graspable ones, also in this case regardless of presentation modality. 

Taking together the results of [56] and the present ones, one may argue that motor responses are fine-tuned with the motor representation of the processed stimuli, since non-graspable natural objects, as well as graspable tools, do not modulate motor responses in the same direction of graspable natural objects. 

The interference effect found in the behavioral experiment (Experiment 1) was replicated in the MEG experiment (Experiment 2), where participants were requested to perform the same go/no-go task while assessing the cortical beta rhythm. In Experiment 2, motor responses to natural graspable objects confirmed to be slower than those given to tools, thus further supporting the notion that tools and natural graspable objects have a different representation within the motor system. Coherently, beta rhythm, as revealed by MEG, had a weaker decrease during the processing of natural graspable objects as compared to tools. A suppression of beta rhythm (the so-called ERD), normally recorded in motor/premotor areas, occurs when these areas are involved in the actual execution of an action or, at a less degree, when individuals observe or imagine an action [72,87]. In other words, our results show that, during the processing of natural stimuli, ERD is weaker than during the processing of tools, thus suggesting that the motor system is less prompt to give a motor response. This weaker suppression appears the neurophysiological correlate of the interference effect (i.e., the slowing down of hand motor responses during the processing of natural graspable objects, either verbally or pictorially presented) obtained in the behavioral task. 

It is worth stressing that converging results also come from very few fMRI studies showing shared neural substrates activation during the processing of nouns and visually presented objects [88,89,90], thus supporting further the view of a common semantic system for both nouns and their corresponding [91,92,93]. Similar results were obtained during behavioral, neurophysiological, and MEG studies where participants were asked to process observed hand-actions and verbs expressing actions in the same category, either taken separately or combined [63,64,65,66,67,71,74,94,95].

As far as observed natural objects, the present results are in keeping with the current literature [2,16,20,24], showing that the dorsal stream is involved when participants observe natural graspable objects, as the relevant features of these objects are the motor ones. However, the present results show that a similar modulation of behavioral motor responses and beta rhythm occur also for verbal labels referring to the same object category, thus suggesting that the dorsal stream was similarly involved independent of the presentation modality. This evidence does not fit with the approach claiming that the conceptual knowledge about an object is represented in semantic hubs distinct from the brain areas where object properties are coded [13,16,20], being the semantic hubs widely coinciding with posterior inferior parietal lobule (including the angular gyrus, IPL), middle temporal gyrus, fusiform and parahippocampal gyri, dorsomedial prefrontal cortex, IFG, PMv, and posterior cingulate gyrus [13,21]. 

Some authors consider the recruitment sensorimotor areas within the dorsal stream during language processing as due to late effect related to the spread of activity of top-down cognitive processes most likely occurring in higher order areas involved in object identification [10]. In other words, they consider the recruitment of these areas as a side effect of the activation of distinct cognitive areas crucial for the semantics. This view claims that ‘‘sensory and motor information color conceptual processing, enriches it and provides it with a relational context’’. Since this top-down additional process requires time, we tend to rule out this explanation, since processing of our stimuli is time locked at about 150 ms from stimulus presentation, a time window which rules out the occurrence of motor system recruitment as a side effect of upstream cognitive processes [64,65,71,95,96,97,98]. 

A second point of interest is the evidence that observed graspable tools and nouns referring to this object category do not modulate the activity of the motor system in the same manner as natural objects do. As we stated in the introduction, tools are a special class of graspable objects that imply special hand–object interactions. Manipulation of tools is mainly devoted to a specific use (i.e., functional, [28]) rather than to a simple structural grasping that is used for natural graspable objects. Since structural grasping actions that we can act upon natural objects, are likely shared also with other species (even phylogenetically far from human primates), and have a distinct cortical representation [28,29,53], in this context we refer to these grasping actions as “ecological” ones.

Despite there is a general debate on the use of tools in monkeys [99,100] there is no doubt that only humans possess specialized neural mechanisms allowing them to understand the functional properties of tools. Moreover, only humans have the capacity to generalize the use of a tool in different contexts and to build up new tools depending on their needs. A so fine developed ability seems to have its neural basis in the left IPL that appears as a specific sector only evolved in humans, distinct from monkey grasping regions [54,101]. Within the dorsal stream, this area is referred to as ventro-dorsal sector [27,28,29,102]. A further consideration that supports the notion that the use of tools is exclusive for humans comes from clinical neurology. Apraxia is a syndrome where patients may lose the capacity to use tools properly [103,104,105]. Apparently, there is no counterpart of apraxia syndrome in the monkeys [106]. If one accepts the notion that the semantic of objects is coded where the objects are motorically represented, then processing tools should imply the involvement of the corresponding brain sector in the ventro-dorsal circuit. The results of the present study are in line with this view. Tools, whatever the modality of presentation, did not modulate the motor responses as well as beta rhythm, like natural objects did. This evidence may be explained by the fact that participants used a very simple motor act to provide their responses (pushing a button), an action represented in the circuit devoted to interactions with natural objects (ecological grasping actions) rather than in the circuit devoted to the use of tools. A similar distinction was revealed by using TMS [107] in a study where motor evoked potentials (MEPs) were obtained during the observation of graspable and non-graspable natural objects and tools, respectively. Results showed that MEPs elicited by natural graspable objects had a less amplitude than those elicited by graspable tools, again suggesting that a different circuit and a different sector of premotor/motor cortex was involved in processing these two categories of objects.

One could argue that tools nouns did not affect motor responses and beta rhythms in the present experiment because, as foreseen by current literature, nouns are processed in semantic hubs. However, if one assumes that nouns are coded in specific semantic hubs, then the nouns of tools as well as the nouns of natural objects should be coded in these semantic hubs and, consequently, should not modulate the activity of motor areas. In other words, one should expect similar motor responses as well as a similar modulation of beta rhythm when processing nouns referring both to natural graspable objects and tools. The present data, showing that only nouns of natural graspable objects modulate the activity of areas devoted to ecological grasping, further support the notion that the neural substrates of semantics processing overlap with those where the most relevant features of an object are experienced. 

If semantics is coded in the areas where objects are motorically represented or perceptually experienced, then it remains to explain the role of the higher order areas that several authors consider as the actual semantic hubs [13,16,20,108]. Beyond language processing, these areas have been involved in different tasks. Some of them also constitute the nodes of the so-called “default-mode” network, a set of functionally interconnected regions that are consistently modulated during demanding cognitive tasks [109,110] or during social cognition tasks [111,112,113]. As for prefrontal cortex areas, they have been involved in working memory tasks [114] as well as in the re-organization and recall of simple and well-known motor acts in novel actions [115,116,117]. Finally, the IFG, including Broca’s region, is known to be endowed with hand motor representations and has a role in speech production as well as lip [118,119,120]. We forward that the recruitment of these areas during nouns processing and conceptualization, rather than related to semantics, is better explained if we assume that they may contribute to contextualize the processed words, to express how demanding is their processing and, most likely, how much they are related to our life experiences and personal beliefs.

## Figures and Tables

**Table 1:** Descriptive statistic of behavioral study (Experiment 1).

	Noun			Image		
	Mean (ms)	Standard Deviation (ms)	Standard Error (ms)	Mean (ms)	Standard Deviation (ms)	Standard Error (ms)
Natural	708	80.91	15.57	720	94.61	18.21
Tool	666	76.03	14.63	686	93.27	17.95

## Data Availability

All custom scripts and data contained in this manuscript are available upon request from the corresponding author, Giovanni Buccino (buccino.giovanni@hsr.it).

## Supplementary Materials

The following are available online at https://www.mdpi.com/article/10.3390/brainsci12010097/s1, Table S1: Appendix—Stimuli used in Experiment 1 and 2.
