# Supplementary material for: The Semantics of Natural Objects and Tools in the Brain: A Combined Behavioral and MEG Study
Source: Brain Sci. 2022 Jan 12;12(1):97. doi: 10.3390/brainsci12010097 (PMC8774003; doi:10.3390/brainsci12010097)
Supplement: Supplementary file 1 [file brainsci-12-00097-s001.zip › brainsci-1461760-supplementary.pdf]

Supplementary Material - Stimuli used in Experiment 1 and 2

**nouns**	**translation**	**images**	**category**
Bottiglia	bottle	axe	tool
Bottone	button	needle	tool
Chiave	key	stick	tool
Coltello	knife	pen	tool
Cucchiaio	spoon	ball for bocce game	tool
Forbici	scissors	match	tool
Forchetta	fork	cudgel	tool
Maniglia	handle	iron	tool
Ombrello	umbrella	hairpin	tool
Martello	hammer	handbrake	tool
Pettine	comb	knob	tool
Pinza	plier	crank (coffee grinder)	tool
Rasoio	razor	mace	tool
Rubinetto	tap water	clothespin	tool
Sapone	soap (bar of soap)	sea shovel	tool
Scopa	broom	table tennis racket	tool
Tappo	stopper	spatula	tool
Telecomando	remote control	brush	tool
Telefono	phone	sponge	tool
Zip	zip	folding fan	tool
Bulbo	flower bulb	banana peel	natural
Pigna	pine cone	coal	natural
Bocciolo	bud	sea shell	natural
Corteccia	bark	coral	natural
Foglia	leaf	diamond	natural
Fossile	fossil	flower	natural
Cuoio	leather	piece of clay	natural
Granello	granule	acorn	natural
Neve	snow	bone	natural
Paglia	straw	pearl	natural
Pepita	nugget	petal	natural
Picciolo	petiole	bean pod	natural
Pietra	stone	feather	natural
Ramoscello	twig	root	natural
Guscio	shell	stone	natural
Sabbia	sand	ear of corn	natural
Scorza	zest	icicle	natural
Seme	seed	timber	natural
Stelo	stem	musk	natural
Sughero	cork	hairs	natural
